# Independent verification of vendor‐issued dosimetric data for ^106^Ru brachytherapy using diode detectors with traceability to external beam standards for absorbed dose to water

**DOI:** 10.1002/mp.70381

**Published:** 2026-03-29

**Authors:** Simon Dahlander, Ilias Billas, Thorsten Sander, Graham Bass, Linda Persson, Åsa Carlsson Tedgren

**Affiliations:** ^1^ Department of Oncology Pathology Karolinska Institute Stockholm Sweden; ^2^ Department of Nuclear Medicine and Medical Physics Karolinska University Hospital Stockholm Sweden; ^3^ National Physical Laboratory Teddington UK; ^4^ Swedish Radiation Safety Authority Stockholm Sweden; ^5^ Department of Health, Medicine and Caring Sciences Linköping University Linköping Sweden

**Keywords:** dosimetry, Monte Carlo, Ru‐106 brachytherapy, traceable metrology

## Abstract

**Background:**

Independent verification of the manufacturer‐provided dosimetry data for ^106^Ru ophthalmic brachytherapy applicators is crucial for safe and accurate treatment, yet a standardized, traceable method for clinical absolute dosimetry has been lacking.

**Purpose:**

This work establishes a complete framework for traceable absorbed dose to water measurements of ^106^Ru eye plaques in absolute units of Gray, complementing the high‐precision BetaCheck‐106™ setup with a robust detector calibration methodology independent of the source manufacturer.

**Methods:**

Three microSilicon diode detectors were calibrated in traceable ^60^Co and 6 MeV electron reference beams. Depth‐dose measurements for four CCB‐type ^106^Ru plaques were performed in water using the BetaCheck‐106™ setup. Monte Carlo (MC) simulations were employed to calculate depth‐dependent beam quality correction factors $k_{Q,Q_{0}}$ which account for the detector's response in the ^106^Ru field relative to the calibration beams. The method was validated against measurements performed at the National Physical Laboratory (NPL) using alanine dosimetry. MC simulations were also used to investigate the water‐equivalence of the NPL alanine/PMMA phantom setup.

**Results:**

The MC‐calculated correction factors for the diodes showed a significant depth‐dependence, underscoring the necessity of such corrections in the steep ^106^Ru depth‐dose gradient. The dose rates determined with the calibrated diodes were in agreement with the NPL alanine results. Both methods yielded dose rates systematically lower than those provided in the manufacturer's certificates, though generally within the stated uncertainties. The MC simulations revealed substantial non‐water equivalence correction factors for the alanine/PMMA phantom, highlighting the advantage of direct measurements in water.

**Conclusions:**

We present a novel, comprehensive methodology for independent and traceable absolute dosimetry of ^106^Ru applicators. By combining a dedicated water phantom setup with diode detectors calibrated against external beam standards and MC‐derived correction factors, this framework empowers clinical users to perform robust verification measurements, filling a critical gap in the quality assurance of ocular brachytherapy.

## 1 INTRODUCTION

Uveal melanoma (UM) is the most common primary intraocular malignancy in adults, and the most commonly used eye‐preserving treatment for UM is brachytherapy.^1^ Brachytherapy for UM utilizes metallic applicators, referred to as “eye plaques”, often made of silver or gold, which house a radionuclide on their concave side. The treatment involves surgically attaching the plaque to the sclera at the tumor site and leaving it in place to irradiate until the desired absorbed dose has been delivered to the tumor, which usually takes several days. The plaques either use a distributed source or an array of brachytherapy seeds. In Europe, plaques with distributed ^106^Ru sources are most commonly used.^2^

^106^Ru is a beta‐emitter with an average emission energy of 1.41 MeV, resulting in a short electron range in water with a very steep absorbed dose gradient, about 2% per 0.1 mm at the applicator surface.^3^ This, in combination with the special geometry of the source (distributed over a concave surface), has made the dosimetry of this applicator difficult. The ^106^Ru plaques are exclusively manufactured by Eckert & Ziegler BEBIG GmbH, Berlin, Germany. Each plaque comes with a certificate containing the measured absorbed dose rate to water from the central axis of the plaque surface up to a distance of 10 mm from the surface, as measured by the vendor's in‐house developed plastic scintillator. This plastic scintillator is calibrated using a so‐called “reference plaque”, with a surface dose rate measured at the accredited dosimetry calibration laboratory (ADCL) of the University of Wisconsin Medical Radiation Research Center (UWMRRC). The surface dose rate measurements are done using a specially developed convex windowless extrapolation chamber.^4^ There is currently no standard procedure for clinics to independently verify this data. As a result, many clinics use the vendor‐issued dosimetric data without independent verification.^5^, ^6^ In order to reach the same high safety standards present in other forms of radiotherapy, a reliable method for dosimetric verification needs to be developed.

This study aims to establish a complete framework for the traceable dosimetry of ^106^Ru eye plaque brachytherapy, enabling measurements of absorbed dose to water in absolute units of Gray using direct reading detectors. This framework integrates two critical components: a dedicated measurement system and a traceable calibration method. The first component, a compact water phantom detector‐applicator setup (BetaCheck‐106), was evaluated in Dahlander et al. (2026)^7^ for its high precision and reproducibility in measuring ^106^Ru depth‐dose distributions. The present work provides the second component: a robust methodology for the absolute calibration of direct‐reading detectors. To achieve this, four CCB eye plaques were measured with three PTW microSilicon diode detectors (PTW GmbH, Freiburg, Germany) using the BetaCheck‐106 setup. Silicon diodes were selected for their high sensitivity relative to their active volume, making them well‐suited for precise dose measurements in small fields.

The silicon diodes were calibrated using external beam radiation fields, with calibration factors for absorbed dose to water determined in both a ^60^Co beam and a clinical 6 MeV electron beam. Correction factors for measurements in the ^106^Ru plaque field were derived using Monte Carlo (MC) simulations. To validate the proposed method, absorbed dose measurements for the same four plaques were also performed using alanine dosimetry at the National Physical Laboratory (NPL) in Teddington, United Kingdom. The NPL alanine protocol has been serving hospitals for more than 20 years, but it has not been previously published. This paper will therefore provide a detailed outline of the method, including a MC simulation of the setup.

The depth‐dose rate to water measurements obtained with the silicon diode detectors were compared to the results of the alanine measurements, as well as with the vendor‐supplied dosimetric data.

## 2 METHODS AND MATERIALS

### 2.1 Dosimetry formalism

The dosimetry formalism used in this work is based on calibration of the detectors in an external beam field with traceability to external beam standards for the quantity absorbed dose to water, following the IAEA TRS‐398 protocol.^8^ The formalism described has been applied in previous studies to measure ^192^Ir brachytherapy photon fields with lithium formate electron paramagnetic resonance (EPR) detectors,^9^ LiF TLD^10^ and microDiamond detectors.^11^ A similar methodology, using a now outdated protocol and without detailed MC simulation of the plaque‐detector geometry, was used by Lax^12^ for measuring ^106^Ru with a silicon diode detector.

The measurement of the absorbed dose to water $D_{w,Q}$ in a beam quality Q is given by

(1) $$D_{w,Q}=M_{Q}\;\cdot N_{D,w,Q_{0}}\cdot k_{Q,Q_{0}}$$

where $M_{Q}$ is the reading of the detector in beam quality Q, $N_{D,w,Q_{0}}$ is the calibration coefficient of the detector in terms of absorbed dose to water in the reference beam quality $Q_{0}$, and $k_{Q,Q_{0}}$ is the beam quality correction factor given by

(2) $$k_{Q,Q_{0}}=\frac{{\left[D_{w}/{\bar{D}}_{det}\right]}_{Q}\;}{{\left[D_{w}/{\bar{D}}_{det}\right]}_{Q_{0}}}\;\frac{R_{Q_{0}}}{R_{Q}}$$

where ${\bar{D}}_{\det,\;Q}$ is the average absorbed dose to the active volume of the detector at the point of measurement in beam quality Q, and $D_{w,Q}$ is the corresponding absorbed dose to water at the point of measurement in the absence of the detector. ${\bar{D}}_{\det,\;Q_{0}}$ and $D_{w,Q_{0}}$ are absorbed doses to the active volume of the detector and a point in water, respectively, under calibration conditions in beam quality $Q_{0}$. The absorbed‐dose energy‐response of the detector, that is, the quotient ${\left[D_{w}/{\bar{D}}_{\det}\right]}_{Q}$ and ${\left[D_{w}/{\bar{D}}_{\det}\right]}_{Q_{0}}$, can be calculated using MC methods, see Section 2.6. The intrinsic energy response of the silicon diode detector $R_{Q}=M_{Q}\;/{\bar{D}}_{\det,Q}$ (and equivalently for $R_{Q_{0}}$) describes the physical signal generation and its collection efficiency in the detector. For this work, two calibration fields $Q_{0}$ are investigated (^60^Co and 6 MeV). It is further assumed that $R_{Q_{0}}/R_{Q}\equiv 1$, thus disregarding a potential intrinsic energy difference for the detectors between the calibration beam $Q_{0}$ and the measurement beam Q. This assumption will be further discussed, and accounted for in the uncertainty budget.

The absorbed dose to water is to be determined as a function of depth, *z*, from the center point of the ^106^Ru plaque surface. Hence, the beam quality correction factor needs to be determined as a function of depth $k_{Q,Q_{0}}$(z).

### 2.2 Diode detectors and setup equipment

The microSilicon diode detector was chosen based on the results in Dahlander et al. (2026).^7^ This detector has a nominal active volume of 0.03 mm^3^, with a radius of 0.75 mm and a thickness of 18 µm. For brevity, the microSilicon detector will be referred to as µSi, and the three microSilicon detectors used will be denoted µSi‐1, µSi‐2 and µSi‐3.

The setup equipment and measurement methodology was described and evaluated in detail in Dahlander et al. (2026),^7^ where it's precision and limitations with different detectors (including the µSi) were investigated.

### 2.3 Diode calibration

As mentioned in Section 2.1, the calibration procedure follows the IAEA TRS‐398 protocol.^8^

#### 2.3.1 ^60^Co calibration

The µSi detectors were calibrated for absorbed dose to water in a ^60^Co radiation therapy field at the Swedish National Metrology Laboratory for Ionizing Radiation (Swedish Radiation Safety Authority, SSM), traceable to Bureau International des Poids et Mesures (BIPM). Measurements were conducted in a 300 mm × 300 mm × 300 mm water phantom.

#### 2.3.2 Electron beam calibration

The µSi detectors were also cross‐calibrated in a 6 MeV clinical electron beam from a Varian TrueBeam linear accelerator at the Karolinska University Hospital. The 6 MeV field output was traceable to an ion chamber calibrated in the same ^60^Co source as used for the ^60^Co calibration (see 2.3.1). The energy selected was the lowest available electron energy from the TrueBeam. The detectors were placed inside a 500 mm × 500 mm × 410 mm Blue Phantom^2^ water phantom (IBA Dosimetry GmbH, Schwarzenbruck, and Germany) and irradiated. The detectors were positioned so that the surface of their active volume coincided with the reference depth *z_ref_*.

### 2.4 Measurements with diode and prototype setup‐equipment

Measurements were performed on four BEBIG CCB type ^106^Ru eye applicators using the three diode detectors. Dose rates were recorded at 1 mm intervals, ranging from 2 to 10 mm in water‐equivalent distance, measured from the surface of the detector's active volume to the center of the plaque surface. Based on manufacturer specifications for photon measurements, the active volume of the detector was assumed to be positioned 0.9 mm beneath the detector surface.

Although the manufacturer specifies an effective depth of 0.3 mm for electron field measurements, MC simulations using this value resulted in larger correction factors than correction factors using the 0.9 mm effective depth assumption. This suggests that the active volume specification for photon measurements provides a better fit for these experiments. Consequently, the 0.9 mm positioning was adopted. Correction factors $k_{Q,Q_{0}}$(z) were calculated using this same positioning, ensuring that any deviations from the true effective measurement point were accounted for, provided that the detector setup matched the MC simulations.

To facilitate direct comparison with manufacturer‐provided depth‐dose rate curves, the measurement procedure replicated the manufacturer's approach. For each plaque, measurements were first taken from 2 to 10 mm in depth. At each distance, ten measurements were recorded, with acquisition times ranging from 10 s at 2 mm to 60 s at 10 mm, as the detector signal weakened with depth. Following this sequence, the electrometer was zeroed, and measurements were repeated in reverse order, from 10 mm back to 2 mm. This procedure helped identify and mitigate any systematic bias due to potential background noise accumulation over time. Background measurements were taken before and after zeroing the electrometer, and their average was subtracted from the plaque measurements to correct for background signal contributions.

### 2.5 Alanine measurements

**FIGURE 1:**
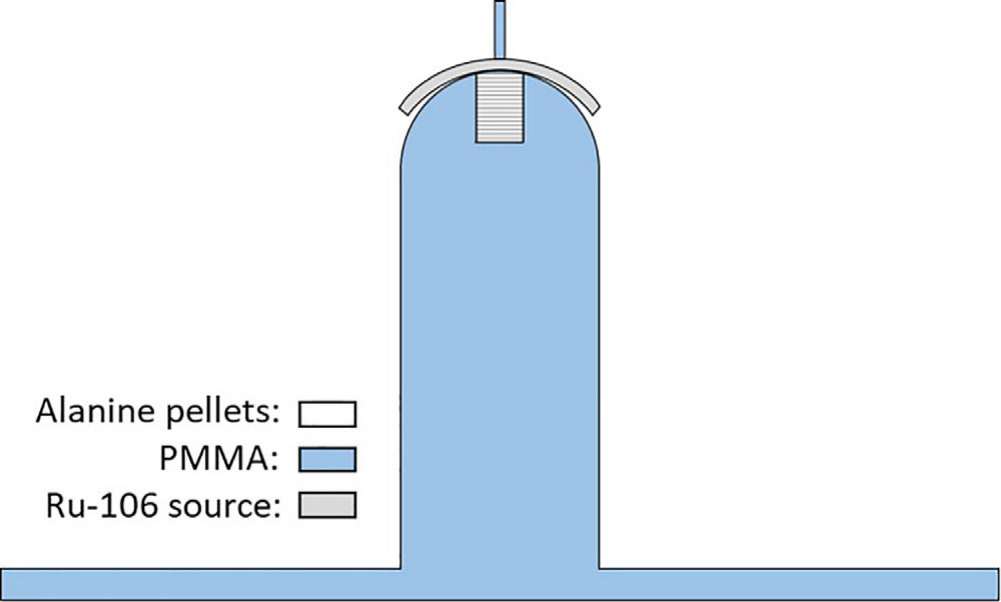
A stack of 11 alanine pellets mounted on a curved PMMA phantom.

The experimental setup at NPL involved irradiating thin alanine pellets, each measuring 0.5 mm in thickness and 5 mm in diameter, using the same CCB plaques as in the µSi diode detector measurements. A stack of 11 alanine pellets was mounted on a curved PMMA phantom (Figure 1). Each ^106^Ru eye plaque was positioned on top of the phantom, ensuring that the center of its active area was in proximity to the topmost alanine pellet^i^. The plaques remained in place for approximately 5 days during irradiation to ensure a sufficient dose for achieving low uncertainties in the alanine readings. To verify the setup repeatability, the exposure using one of the CCB type sources was repeated three times.

More information regarding alanine dosimetry can be found in the literature.^13^, ^14^ Briefly, the amino acid alanine is a chemical detector and when irradiated it forms stable free radicals. The concentration of these radicals correlates directly with the absorbed radiation dose and can be quantified through EPR spectroscopy. In this work, alanine has been calibrated in terms of absorbed dose to water in a ^60^Co beam, traceable to the NPL primary standard for absorbed dose to water, a graphite calorimeter.

The absorbed dose to water in a ^106^Ru source quality, $D_{w,Q_{Ru}}$, was measured by using alanine (al), and similarly to equation 1, is defined as:

(3) $$D_{w,Q_{Ru}}=M_{al,Q_{Ru}}\;\cdot N_{D,w,Q_{0},al}\cdot k_{Q_{Ru},Q_{0}}$$

where, $M_{al,Q_{Ru}}$ is the alanine EPR signal, $N_{D,w,Q_{0},al}$ is the alanine calibration coefficient for the ^60^Co beam energy, $k_{Q_{Ru},Q_{0}}$ is the beam quality correction factor that adjusts the alanine calibration from ^60^Co to ^106^Ru. The approach used in Equation 2 was also applied to define $k_{Q_{Ru},Q_{0}}$:

(4) $$k_{Q_{Ru},Q_{0}}=\frac{{\left(D_{w}/D_{al}\right)}_{Q_{Ru}}}{{\left(D_{w}/D_{al}\right)}_{Q_{0}}}\;\cdot \frac{R_{al,Q_{0}}}{R_{al,Q_{Ru}}}$$

where,

(5) $$R_{al,Q_{Ru}}=\frac{M_{Q_{Ru}}}{D_{al,Q_{Ru}}}\;\mathrm{and}\;R_{al,Q_{0}}=\frac{M_{Q_{0}}}{D_{al,Q_{0}}}\;$$

The absorbed dose to water, $D_{w}$, and to alanine, $D_{al}$, for beam qualities $Q_{Ru}$ and $Q_{0}$ can be calculated using MC simulations, as discussed in Section 2.6.3. It is assumed that the relative intrinsic sensitivity of alanine, $\frac{R_{al,Q_{0}}}{R_{al,Q_{Ru}}}$, is unity with an uncertainty of 0.5%^ii^, implying that ^106^Ru and ^60^Co energies have the same effect on alanine's intrinsic sensitivity.

### 2.6 MC simulations

The MC simulations were done using the general purpose main program penEasy^16^ for the PENELOPE‐2018^17^, ^18^, ^19^ MC system. The built‐in geometry package PENGEOM was used to define detectors and experimental setup geometries using quadratic surfaces.

For all calculations, the electron cutoff energy was 10 keV and the photon transport cutoff energy was 1 keV in all regions. The threshold kinetic energy for inelastic collisions (WCC) was 10 keV and the threshold energy for radiative collisions (WCR) was 1 keV. The elastic collision parameters (C1 and C2) were 0.05 in the scoring volume and nearby regions and 0.1 in other regions.

The simulations used two radionuclide files, ^106^Rh for the eye plaques and ^60^Co for one of the microSilicon diode calibration fields, which were obtained from the Laboratoire National Henri Becquerel (LNHB) database.^20^ The 6 MeV electron field for the diode calibration was generated using a phase space file of the Varian Clinac 6 MeV electron field, assuming that this would be equivalent to the TrueBeam field. The ^60^Co calibration field for the alanine pellets was simulated using phase space files of the NPL ^60^Co field.

#### 2.6.1 Simulations of plaque in water

The energy deposition from the ^106^Ru eye plaque in a small water volume in water was scored. The water volume used as the detector material was defined as a cylinder with a thickness of 18 µm and a radius of 0.75 mm, which matches the active volume of the µSi detector.^21^

In order to verify this setup, the resulting dose to water $D_{w}$ (in units of pGy/decay) was compared to the results of simulations previously done by Sommer et al. (2017).^22^

#### 2.6.2 Diode correction factors

The MC calculations used to find $k_{Q,Q_{0}}$ scored average energy imparted to the active volume of the detector, which was then used to find the average absorbed dose to the detector ${\bar{D}}_{det}$. The energy imparted to a corresponding water volume in the absence of the detector was scored and used to derive the absorbed dose to water $D_{w}$. This was done for the eye plaque field and the calibration field, the beam qualities of the two fields are referred to as Q and $Q_{0}$, respectively. The detector was simulated at positions equal to those used for the measurements and the correction factors $k_{Q,Q_{0}}$ were calculated from Equation 2.

The plaque was modeled according to specifications in ICRU Report 72^23^ and the µSi detector was modeled using blueprints obtained under a non‐disclosure agreement from the vendor. As previously mentioned, the 6 MeV electron field was generated with a phase space file, while the ^60^Co field was instead created using a radionuclide file and custom geometry, replicating the experimental geometry described in Section 2.3.1.

#### 2.6.3 Alanine setup

**FIGURE 2:**
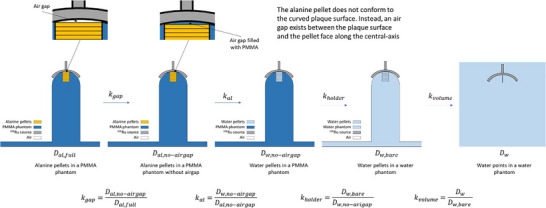
MC calculated perturbation correction factors to determine dose to water at a point in water.

A series of MC simulations were performed to calculate the perturbation correction factors that influence the measurement of absorbed dose to water in the NPL alanine setup for ^106^Ru eye plaques. The perturbations are shown in Figure 2 and correspond to four factors that will correct the dose to water realized with alanine in a full scatter phantom to dose to water at a point in water. These include the air gap between the topmost pellet and the source surface, $k_{\mathrm{gap}}$, the medium perturbation correction factor, $\;k_{\mathrm{al}}$, the holder perturbation correction factor, $k_{\mathrm{holder}}$, and the volume averaging perturbation correction factor, $k_{\mathrm{vol}}$. The product of all these factors is equal to the numerator of Equation 4.

The calculation chain of perturbation correction factors is chosen arbitrarily but consistently as follows. The first geometry represents the full setup, including the source, the stack of alanine pellets and the holder, as shown in Figure 2. In the second geometry, the air gap is filled with PMMA. The third geometry replaces the alanine with water and the fourth geometry replaces the PMMA holder with water. The final geometry is a water point with a radius of 0.75 mm and a thickness of 18 µm (representing the active volume of the PTW 60023 µSi diode) in a water phantom large enough to provide electron equilibrium.

The stack of alanine pellets was constructed as a cylinder with radius 2.5 mm and height 7.04 mm (0.44 mm × 16 pellets). Dose was scored in 16 cylindrical bins on this volume, each bin equaling one pellet, using the penEasy Cylindrical Dose Distribution tally. Dose to water $D_{w}$ was scored as specified in section 2.6.1.

### 2.7 Uncertainty budget

The uncertainty analysis presented here follows the Joint Committee for Guides in Metrology (JCGM) Guide to the Expression of Uncertainty in Measurement (JCGM 2008). Uncertainties evaluated by statistical analysis are grouped as type A and the rest are grouped as type B. These are added in quadrature to give a combined standard uncertainty with coverage factor k = 1.

#### 2.7.1 Diode measurements

The quoted relative standard uncertainties for the diode measurements are shown in Table 1. Details regarding the determination of the individual uncertainty components of the ^106^Ru measurements can be found in Dahlander et al. (2026),^7^ specifics regarding uncertainty components of the traceability are provided in the appendix.

**TABLE 1:** Uncertainty budget for diode measurements.

		Relative standard uncertainties		
Uncertainty component		Type A (%)		Type B (%)
**^106^Ru measurements**				
Half‐life of ^106^Ru				0.04
Half‐life resolution (days)				0.05
Electrometer correction				0.1
Temperature correction				0.1
Recombination		–		–
Long term stability microSilicon		0.1		
Measurement Repeatability		0.3–0.8		
Setup Reproducibility		0.21		
**Traceability (^60^Co)**				
N*_D_*_,w_ (^60^Co)				0.5
k_QQ0_		0.2		0.5
R_QQ0_, Difference in silicon diode intrinsic sensitivity				1.0
**Traceability (6 MeV)**				
N*_D_*_,w_ (6 MeV)				1.1
k_QQ0_		0.3		0.5
R_QQ0_, Difference in silicon diode intrinsic sensitivity				1.0

	Co‐60		6 MeV	
	2 mm	10 mm	2 mm	10 mm
Overall combined relative standard uncertainty	1.6	2.1	2.6	3.1
Expanded relative uncertainty (*k *= 2)	3.2	4.2	5.2	6.2

#### 2.7.2 Alanine measurements

The quoted relative standard uncertainties are shown in Table 2. Details regarding the determination of the individual uncertainty components are provided in the appendix. The overall combined relative standard uncertainty in the absorbed dose to water determined with alanine, was found to be between 2.5% and 3.9% (for 2 to 5 mm depth). The dominant uncertainty component is the setup repeatability and alanine variability, which is depth dependent.

**TABLE 2:** Uncertainty budget for the measurement of absorbed dose to water from a ^106^Ru source measured with alanine.

	Relative standard uncertainties	
Uncertainty component	Type A (%)	Type B (%)
Alanine calibration coefficient in a ^60^Co energy beam		1.2
Half‐life of ^106^Ru (days)		0.2
Time of exposure		0.1
Temperature correction		0.2
Difference in alanine intrinsic sensitivity		0.5
MC correction/conversion factor	0.5	
Setup repeatability and alanine variability	2.2 (2 mm) 2.1 (3 mm) 2.1 (4 mm) 3.6 (5 mm)	
Overall combined relative standard uncertainty in the absorbed dose (*k *= 1)	2.6 (2 mm) 2.5 (3 mm) 2.5 (4 mm) 3.9 (5 mm)	

## 3 RESULTS

### 3.1 Validation of MC models

**FIGURE 3:**
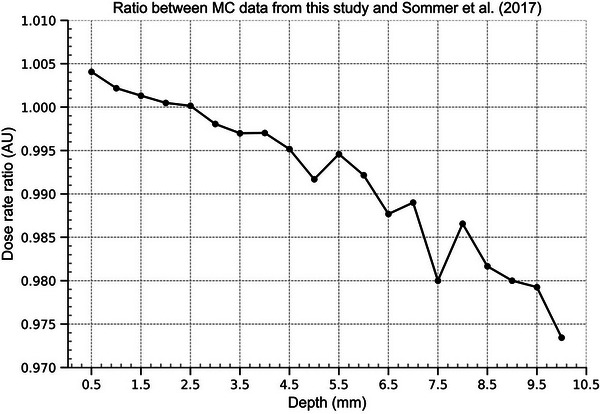
MC calculated dose to water from ^106^Ru eye plaques, ratio between results from this study and the MC study by Sommer et al. (2017).

For the purposes of validating the CCB applicator model, a comparison between the MC results of dose to water from a CCB plaque was made between this study and the GEANT4 MC study by Sommer et al. (2017). The graph in Figure 3 shows the ratio between the two data sets as a function of depth in water. The deviations between the PENELOPE and GEANT4 results change with depth, up to a maximum 2.5 % deviation at 10 mm depth.

**FIGURE 4:**
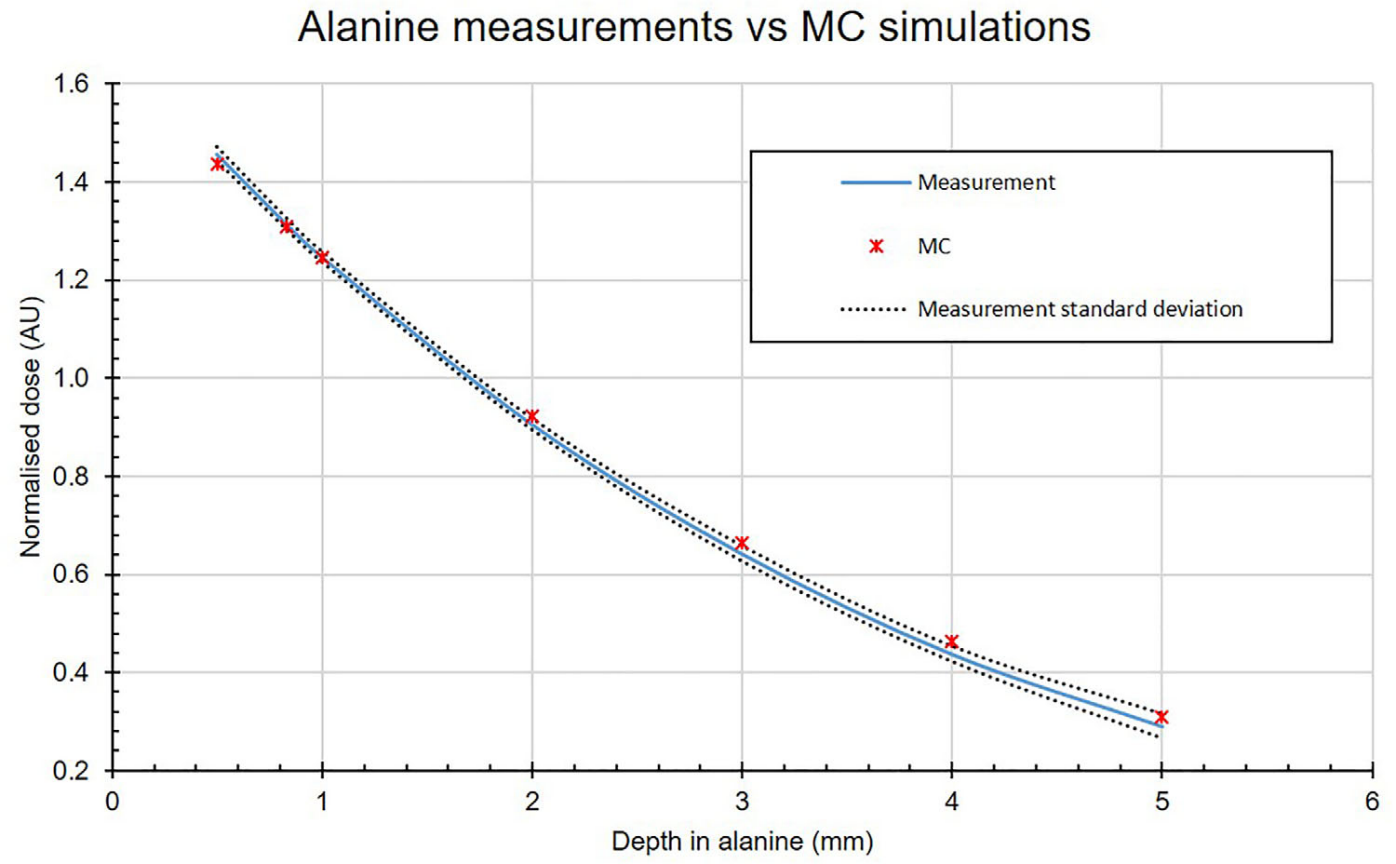
Alanine measurements of 31 CCB sources, measured at NPL since 2018, compared with the MC simulation results.

For the validation of the MC model of the alanine setup, data from 31 CCB sources measured at NPL since 2018 were compared with MC results of $D_{al,full}$ (see Figure 2). Figure 4 shows the comparison between the alanine measured and MC‐calculated depth‐dose distributions. For both the measurement and the MC, the data are normalized. The normalization was done by taking the dose measured (or MC calculated) for each depth and averaging it over all depths, this average over the depths was used to normalize the data to allow for a comparison between MC‐calculated and alanine measured values.

The blue line shows the average of the 31 CCB sources. The agreement between the measured and MC data is within the standard deviation of the measurements (shown with the dashed lines as the standard deviation).

### 3.2 Diode beam quality correction factors, $k_{Q,Q_{0}}$

The $k_{Q,Q_{0}}$ correction factors were calculated for CCB applicators for each measuring distance with ^60^Co and 6 MeV µSi diode calibrations. The resulting factors can be found in Table 3, note that these factors are only valid under the setup‐conditions outlined in the methods section.

**TABLE 3:** Depth‐dependent correction factors for CCB plaque measurements with the two different calibrations.

Depth [mm]	$k_{Q,Q_{0}}$ (^60^Co)	$k_{Q,Q_{0}}$ (6 MeV electrons)
2	1.14	1.01
3	1.13	1.01
4	1.12	1.00
5	1.10	0.98
6	1.09	0.97
7	1.08	0.96
8	1.07	0.95
9	1.06	0.94
10	1.04	0.93

### 3.3 Alanine simulations

**FIGURE 5:**
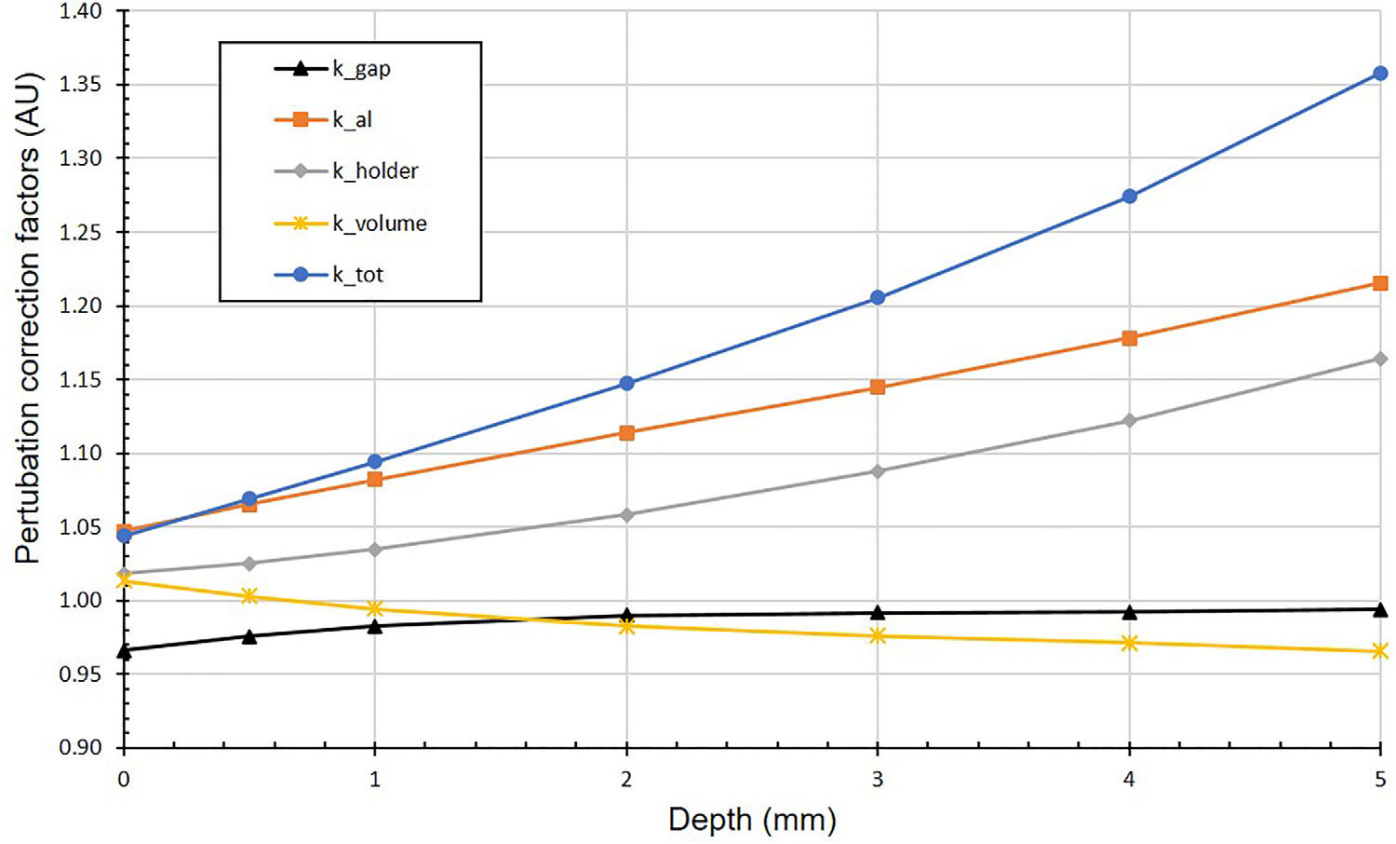
Different perturbation correction factors for the NPL alanine setup.

Figure 5 shows the MC‐calculated perturbation correction factors for the NPL alanine setup for ^106^Ru plaque measurements. The correction factor $k_{gap}$ remains below 1.00 at shallow depths and approaches 1.00 as the depth increases; the factors $k_{al}$ and $k_{holder}$ have a large increase with depth and the factor $k_{volume}$ exhibits a slight decrease with increasing depth.

### 3.4 Dose rate to water measurements

**FIGURE 6:**
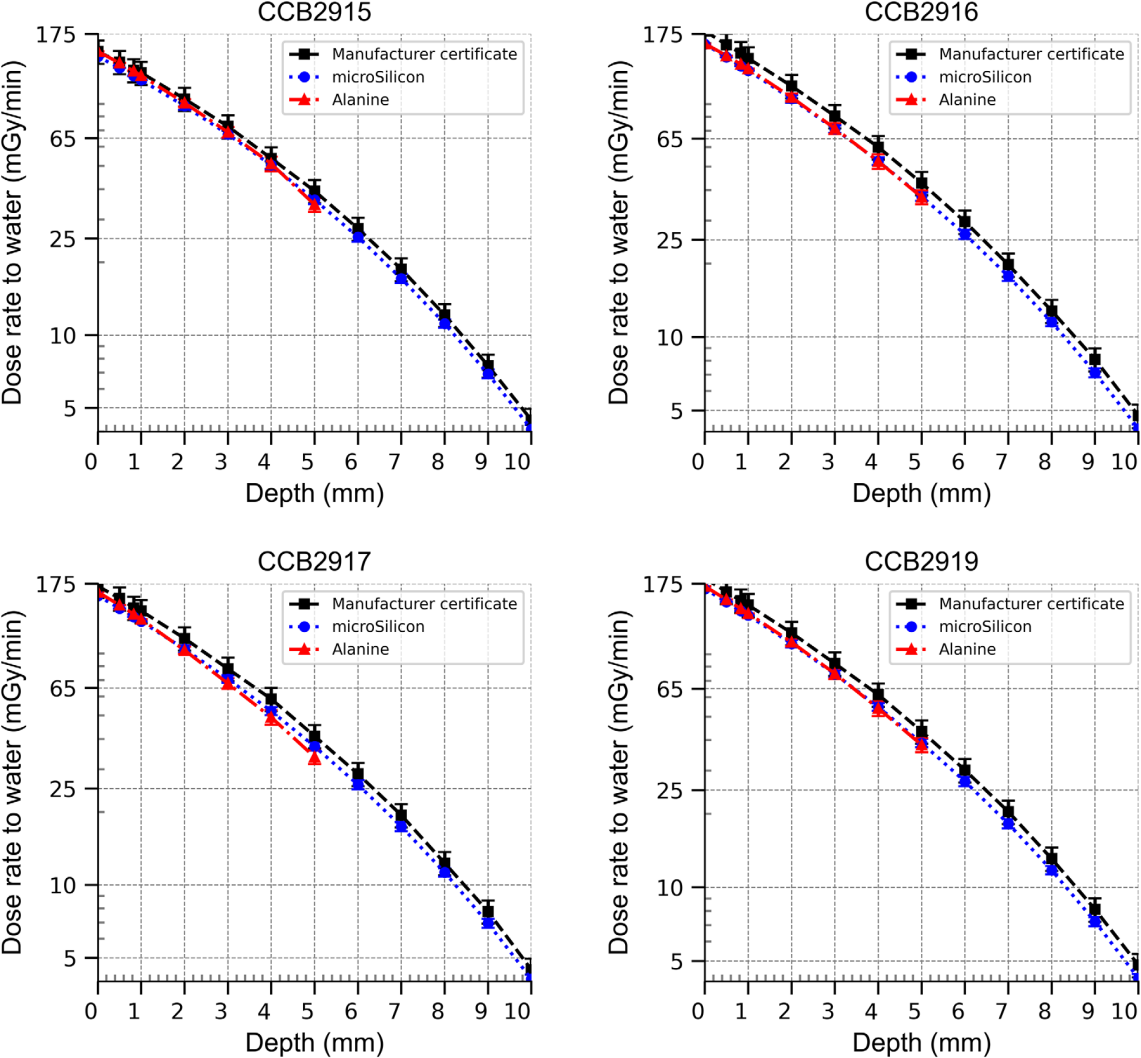
Depth dose curves of the plaques determined from NPL, Karolinska, and the manufacturer. The Karolinska data sets are with ^60^Co calibration. The uncertainty bars correspond to the total expanded uncertainty (*k *= 2). Measurement uncertainties not displayed for extrapolated values.

**FIGURE 7:**
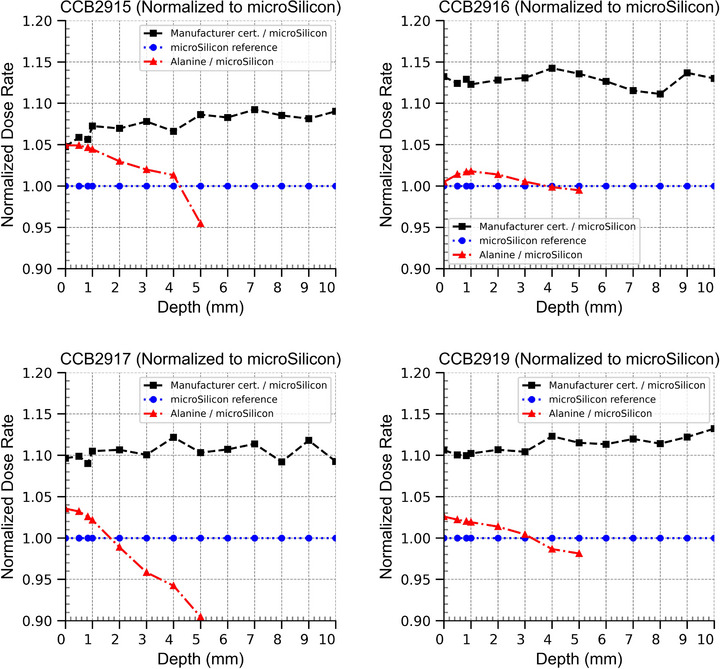
Depth dose curves of the plaques normalized to the Karolinska microSilicon measurements using the ^60^Co calibration.

The dose rates measured for the four plaques can be found in Figure 6, where data have been averaged for all three µSi diodes. Note that diode data below 2 mm depth were extrapolated using polynomial regression. The dose rates measured in this study are consistently lower than what was measured by the manufacturer, both for the alanine and µSi measurements. The manufacturer dose rate measurement has an uncertainty of 11% (*k *= 2) according to the certificate. The difference between manufacturer dose rates and the dose rates from this study can be clearly seen in Figure 7, where alanine and manufacturer dose rates were normalized to the µSi data. The data mostly agree within the combined expanded uncertainties, but there are a few exceptions. The calibration field used for the diodes in Figure 6 was ^60^Co. Using the 6 MeV calibration gave dose rates 0.75% lower than the ^60^Co calibration.

## 4 DISCUSSION

### 4.1 Simulations of bare plaque in water

The findings presented in Figure 3 reveal that the PENELOPE simulations conducted in this investigation yield results that agree with Sommer et al.’s GEANT4 simulations^19^ when measuring energy deposition in the vicinity of the plaque surface. At the maximum distance of 10 mm, the pGy/decay values obtained in this study are approximately 2.5 % lower than those reported by Sommer et al. When Sommer et al. compared the GEANT4 results to relative depth dose profiles obtained from a PENELOPE simulation, the results agreed within a margin of 2.5 %.^22^ This implies that the difference found in this study is of the order expected when comparing GEANT4 and PENELOPE simulations of ^106^Ru plaques. Since the data obtained at greater source distances are subject to greater uncertainty, it is reasonable to expect that perfect correspondence between this study and Sommer et al. (2017) at this depth would be difficult to achieve. Nevertheless, the discrepancy between the two sets of results appears to be systematic rather than random, as the ratio between them steadily decreases with depth (with the exception of the 7.5–8 mm range).

### 4.2 Diode correction factors

The correction factors $k_{Q,Q_{0}}$, presented in Table 3, demonstrate a clear variation with depth for both the ^60^Co and 6 MeV electron calibration fields. This variation underscores the necessity of applying depth‐dependent correction factors when performing dosimetry in a field with a steep dose gradient, such as that of ^106^Ru plaques. These findings also emphasize the need for high accuracy in detector positioning when measuring in this field.

The higher $k_{Q,Q_{0}}$ values observed for the ^60^Co calibration, compared to the 6 MeV electron calibration, are consistent with the fact that lower calibration factors were obtained in the ^60^Co field compared to the 6 MeV field. This implies that there is a greater disparity between the calibration and measurement fields in the ^60^Co calibration compared to the 6 MeV electron calibration. However, it is important to note that the 6 MeV electron field was calibrated using an ion chamber that itself was calibrated in a ^60^Co field, introducing additional steps and uncertainties in that calibration chain. As a result, the total uncertainty budget for the ^60^Co calibration combined with its correction factors is lower than that of the 6 MeV electron calibration, despite the larger magnitude of the correction factors.

### 4.3 Alanine simulations

The close agreement between the measured and MC‐calculated depth‐dose distributions (Figure 4) indicates that the MC models of both the ^10^ ^6^Ru source and the alanine setup in the PMMA phantom are accurate. This validates the use of these MC models for calculating the correction factors discussed in Section 2.6.3, ensuring the reliability of the dosimetric formalism applied in this work.

The MC‐calculated perturbation factors for the alanine setup (see Figure 5) show depth‐dependent variations, exhibiting particularly pronounced changes for the factors relating to the non‐water equivalence of the phantom and detector materials.

$k_{gap}$: Corrects for the presence of the air gap between the ^106^Ru source and the uppermost alanine pellet. At shallow depths the air gap introduces a small dose attenuation due to geometric and scatter effects (Air attenuates less of the electrons compared to PMMA and there is a bigger proportion of backscatter electrons reaching the first pellets. Both of these effects increase the dose when an air gap exists.), leading to a correction factor slightly less than 1.000. As depth increases, the effect of the air gap remains relatively stable or diminishes slightly due to reduced influence of near‐surface scatter and due to the curved surface of the source that directs electrons towards the measurement axis in deeper regions.

$k_{al}$: this factor corrects for the differences in dosimetric response between alanine and water. As depth increases, scattering and energy spectrum variations affect the response of alanine differently compared to water. The energy spectrum softens more significantly at greater depths, causing larger differences between alanine and water responses, which leads to increasing correction factors to account for these differences.

$k_{holder}$: this factor corrects for the presence of the PMMA holder. PMMA and water have different attenuation properties (different density and atomic composition). Replacing PMMA with water reduces the electron attenuation, leading to a higher dose in water, and the $k_{holder}$> 1 accounts for this difference. At greater depths, the influence of material properties becomes more pronounced due to increased scatter and attenuation along the electron path and due to the curved surface of the source that directs electrons towards the measurement axis in deeper regions.

$k_{volume}$: this factor considers the difference between the scoring volumes of microSilicon and alanine detectors and corrects for volume averaging when transitioning to a water point geometry (which has the same dimensions of the microSilicon detector). The $k_{volume}$ moving below unity with depth is consistent with the increased influence of volume averaging effects in regions with a steeper dose gradient. At about 1 mm the point dose is nearly equal to the volume‐averaged dose, meaning no correction is needed at this point. At greater depth, as the radiation field is dominated by scattered electrons and secondary radiation

$k_{tot}$ represent the combined effect of all corrections factors and is dominated by the $k_{al}$ and the $k_{holder}$.

These variations highlight how assumptions regarding the water‐equivalence of alanine and PMMA, which are generally used in external beam radiotherapy, do not apply when measuring in the ^106^Ru beta radiation field.

### 4.4 Dose rate to water measurements

In this study, both the dose rates measured with µSi diodes and alanine pellets were within the uncertainties of the manufacturer‐provided dose rates. However, they were also systematically lower. Several factors could explain this discrepancy.

First, BEBIG's measurements are performed using a plastic scintillator, which may overestimate dose rates due to Cherenkov effects in the optical fibers. The β‐particles emitted by ^10^ ^6^Ru are sufficiently energetic to produce Cherenkov radiation up to the 10 mm measurement depth,^24^ and BEBIG does not apply corrections for this effect. Previous studies have reported that Cherenkov light can contribute between 2%^25^ and 12%–17%^26^ to the signal when measuring ^10^ ^6^Ru applicators. However, this effect is expected to decrease with distance as the β‐particles lose energy, a trend not observed in this study.

Second, the reported distance between the plaque and the plastic scintillator in BEBIG's measurements has varied over time due to uncertainties in the scintillator's material compositioniii. If the effective point of measurement is closer to the surface than assumed, it could lead to systematically higher dose rate measurements.

Third, the heterogeneity of the ^10^ ^6^Ru source distribution on the plaques may contribute to discrepancies. The MC simulations in this study assume a homogeneous activity distribution, but real plaques exhibit variations in source distribution.^27^ Hot spots with activity exceeding the plaque average by up to 25% have been reported.^28^ This underscores the importance of measuring multiple plaques of the same model when studying ^10^ ^6^Ru dosimetry. A MC study by Zaragoza et al.^28^ demonstrated that source heterogeneity has the greatest impact near the plaque surface. Dahlander et al. (2026)^7^ includes an analysis of 20 CCB plaques with regard to their inter‐applicator variability, which complements the findings of this study.

This heterogeneity in source distribution might explain the variation in results between different applicators, something that is particularly visible in the comparison between alanine and µSi detectors in Figure 7. The difference between alanine and µSi measurements is notably larger for CCB2917 compared to the other applicators. This increased discrepancy could be attributed to greater nonuniformity in the CCB 2917 applicator across the alanine irradiation area. Given that the alanine dosimeters have a diameter of 5 mm compared with 1.5 mm for the µSi detectors, alanine measurements are more sensitive to spatial dose variations.

This raises questions about how heterogeneity might affect BEBIG's calibration procedure, which relies on surface dose measurements from a single reference CCB plaque. Further investigation into these factors is needed to better understand their influence on dosimetric accuracy and to improve the consistency of dose rate measurements across different systems.

The microSilicon diode data with the two different calibrations were within 0.75% of each other, with the ^60^Co calibration giving slightly higher values. This shows that both calibration paths are valid options.

## 5 CONCLUSIONS

A new method has been developed for traceable absorbed dose to water measurements of ^10^ ^6^Ru eye plaques in absolute units of Gray, using direct reading detectors. This method utilizes microSilicon diode detectors calibrated in either a ^60^Co field or a clinical 6 MeV electron beam, combined with MC‐calculated correction factors and a novel experimental setup. The dose rates measured with the diodes showed excellent agreement with alanine dosimetry, and both methods yielded results within the uncertainties of the manufacturer‐provided dosimetric data. However, systematic differences were observed, with both the diode and alanine measurements consistently reporting lower dose rates than those provided by the manufacturer.

Additionally, the MC calculations highlight the strong variation in both energy and dose distribution of the ^10^ ^6^Ru field with depth, underscoring the need for careful consideration when measuring depth‐dose curves. This variation further emphasizes the complexity of dosimetry in ^10^ ^6^Ru plaque fields and the importance of accurate correction factors.

Together with the BetaCheck‐106 equipment, evaluated in Dahlander et al. (2026),^7^ this work forms a complete framework for experimental dosimetry of ^106^Ru applicators. Since this method uses direct reading detectors, it is not as practically cumbersome as methods such as TLDs and alanine. It also measures directly in water, avoiding the need for phantom corrections. It is important to note that the $k_{Q,Q_{0}}$ factors presented here are only valid for CCB applicators under the setup conditions outlined in the methods section. For a complete protocol, different $k_{Q,Q_{0}}$ factors would be needed for every available applicator model.

## CONFLICT OF INTEREST STATEMENT

The authors declare no conflicts of interest.

## Footnotes

i Note that due to the flatness of the alanine pellets, an air gap exists between the topmost pellet and the active area. The effect of this gap on the measured dose is corrected by performing MC simulations, as discussed in the subsequent session

ii The uncertainty is supported by the study of McEwen et al. (2020)^15^ and explained in the Appendix subsection “Uncertainty budget alanine”.

iii Private communications with Michael Andrássy at BEBIG

## Uncertainty budget diode

$N_{D_{w}}{(}^{60}\mathrm{Co})$: The calibration certificate from SSM states a relative measurement uncertainty of 0.5% (*k *= 1).

$N_{D_{w}}\left(6\;\mathrm{MeV}\right)$: Derived relative measurement uncertainty from ^60^Co to 6 MeV electrons in accordance with TRS‐398.

$k_{QQ_{0}}$(Type A): The output data files from PENELOPE contain the statistical uncertainty of energy deposition in detector material. The larger such uncertainty (which typically means the uncertainty when scoring energy deposition at 10 mm distance from the applicator) was used.

$k_{QQ_{0}}$ (Type B): We made an effort to account for the difference between our blueprint‐modeled detector geometry and the real detector. The difference in calibration factors between the three µSi diodes were no more than 1.4%, giving us an idea of the inhomogeneity between the detectors. In order to account for this, the sensitive area of the detector was simulated with variations in thickness of ± 5%. The resulting change in $k_{QQ_{0}}$ was 0.3%. We also simulated with different mean excitation values (I‐values) for Si, the generally accepted I‐value 173 eV was simulated with variations of ± 3 eV. This resulted in changes in $k_{QQ_{0}}$ of 0.4%. Combining these two uncertainties in quadrature gives 0.5%. The reason the mean excitation value was chosen for variation is that Wulff et al. (2010)^29^ found that this is the only factor where reasonable variations produced a change in $k_{QQ_{0}}$ greater than 0.1%.

$R_{Q,Q_{0}}$: A previous study^30^ found a 1.9% expanded uncertainty (*k *= 2) in relative intrinsic energy response between ^60^Co and diagnostic x‐ray beam qualities for PTW's previous silicon diode detector, 60017 Diode E. The material compositions of the µSi and Diode E detectors are similar enough that the findings of the previous study should be applicable here.

## Uncertainty budget alanine

The uncertainty associated with the calibration of the alanine dosimeters in the ^60^Co energy beam, and the uncertainty associated with the variation between individual dosimeter pellets have been determined by statistical methods, from the NPL chemical dosimetry laboratory.

The half‐life of ^106^Ru is 371.5 days,^20^ with a standard uncertainty of ± 0.21 days. Based on the dose range received by the alanine, this corresponds to a maximum relative standard uncertainty of 0.1% in the delivered dose.

Time of exposure: Assuming a 1‐min error over a 5‐day exposure leads to a percentage uncertainty of approximately 0.02% in the total exposure time. This represents a very small contribution to the overall uncertainty, given the long exposure duration.

The uncertainty in the dose delivered during alanine irradiation arises from the impact of temperature on alanine's dose‐response. Alanine has a temperature correction factor of 0.15% per degree Celsius. The temperature during irradiation is measured with an accuracy of ± 0.1°C. However, the temperature itself varied by ± 1°C over the exposure period, which resulted in an uncertainty on the dose of 0.15%.

Difference in alanine intrinsic sensitivity: As reported by McEwen et al. (2020),^15^ the average beam quality correction factor for alanine dosimetry across electron energies from 4 MeV to 22 MeV is 1.014, with an associated uncertainty of 0.5%, based on data compiled from published studies. In our analysis, we also included the value of 1.0091 from the 4 MeV listed in Table 2 of McEwen et al. (2020)^15^; however, this does not affect the average result reported for the 6 MeV to 22 MeV range. The mean beta energy of Ru‐106, approximately 1.41 MeV, lies below the range examined in McEwen et al.’s study, but the values presented can still support an informed estimate of the uncertainty associated with alanine's relative intrinsic sensitivity at lower energies.

The 1.014 correction factor accounts for both 1) the differences in energy deposition, electron fluence spectra and stopping powers (as it was determined, in our study, from MC calculations of the dose‐to‐alanine to dose‐to‐water ratios for both ^60^Co and ^106^Ru beams) and 2) the relative intrinsic sensitivity of alanine. Therefore, a conservative uncertainty of 0.5%, as recommended by McEwen et al. (2020), is adopted for the relative intrinsic sensitivity in our analysis.

The uncertainty on the MC correction/conversion factor arises from various factors that impact the dosimetric accuracy when transitioning from the alanine measurement setup to the desired reference conditions (dose to water). Specifically, it accounts for the air gap between the topmost pellet and the source, the PMMA phantom, the alanine density, the volume averaging and the conversion from dose to alanine to dose to water. It ensures that the correction/conversion factor accurately bridges the gap between the alanine measurement setup and the water‐equivalent dose reference.

The setup repeatability and alanine variability uncertainty is the combination of two components:

1. Setup repeatability. Primarily due to angular deviations, such as the tilt between the source and the central perpendicular axis of the PMMA phantom. This uncertainty was estimated based on MC simulations by tilting the source at an angle with respect to the central perpendicular axis, reflecting possible scenario in the measurements.
2. Alanine pellet‐to‐pellet variation. Alanine pellets may exhibit small variations in their physical properties, with mass differences being the most significant contributor. This uncertainty was estimated based on three repeat measurements of the same source, but with different alanine pellets.
